# The relationship between physical exercise and social adjustment in Chinese university students: the sequential mediating effect of peer attachment and self-esteem

**DOI:** 10.3389/fpsyg.2025.1525811

**Published:** 2025-05-13

**Authors:** Zhi Xing, ChunYan Ge

**Affiliations:** ^1^School of Physical Education and Health, Zhaoqing University, Zhaoqing, China; ^2^Department of Sports, Henan Institute of Physical Education, Zhengzhou, China

**Keywords:** physical exercise, social adjustment, peer attachment, self-esteem, university students

## Abstract

Regular physical exercise is well-documented for its advantages that extend beyond physical health, notably influencing mental and social well-being. This study examines the correlation between physical exercise and social adjustment in Chinese university students (*N* = 672), with peer attachment and self-esteem acting as significant mediators. The analysis revealed a significant indirect effect through peer attachment (effect = 0.0376, 95% CI [0.0091, 0.0588]), self-esteem (effect = 0.2101, 95% CI [0.2730, 0.3690]), and a sequential mediation pathway (effect = 0.0055, 95% CI [0.0023, 0.0224]). Physical activity promotes social ties by encouraging peer bonding, which subsequently facilitates social adaptation. Moreover, self-esteem, a crucial element of psychological resilience, mediates the relationship between physical exercise and social adaptation. Importantly, a sequential mediation pathway—peer attachment—self-esteem—social adjustment—was supported, suggesting that peer relationships enhance self-esteem, which in turn improves social adaptation. This ordering aligns with theories emphasizing the influence of social feedback on self-concept (e.g., Reflected Appraisals Theory, Sociometer Theory). These findings underscore the comprehensive advantages of physical activity and support efforts to promote exercise in academic settings to facilitate student well-being and psychosocial development.

## 1 Introduction

Physical exercise (PE) is acknowledged for its many advantages, greatly enhancing both physical health and psychological resilience (Sagatun, 2010; Xiong et al., 2011; Yan et al., 2020; Yolanda et al., 2021). Multiple research has shown that regular physical exercise may reduce anxiety, boost self-esteem, and promote stress management, therefore promoting general well-being (Holley et al., 2011; Penedo and Dahn, 2005). These advantages are particularly relevant for university students, who are undergoing a distinctive transition from youth to maturity. This shift encompasses both academic requirements and substantial social and emotional adaptations necessary for success in the university environment (Arnett, 2000; Chisholm and Hurrelmann, 1995).

Social adjustment, characterized as the capacity to assimilate and adapt to novel social contexts, is essential for university students, who must navigate both academic and social demands in a rapidly changing environment. Prior research underscores the significance of social adjustment for academic performance; yet, few studies have explicitly examined the influence of physical exercise in promoting this adjustment (Ahn and Lee, 2016). Conventional research on physical exercise mostly emphasizes immediate advantages, including enhancements in physical and mental health outcomes—such as better cardiovascular health, diminished depressive symptoms, and augmented cognitive functioning, among others (Holley et al., 2011; Penedo and Dahn, 2005). However, the extensive social advantages of physical exercise, especially in facilitating social adaptation via indirect factors such as peer bonding and self-worth, have garnered little scientific investigation.

Peer connection and self-esteem are two possible mediators that may connect physical exercise to social adjustment. Peer attachment, defined as the emotional and social connections people establish with their peers, is crucial for young adults who increasingly depend on these relationships for emotional support throughout their transition into adulthood (Ainsworth, 1989; Allen, 1999). Robust peer connections provide kids a “safe haven” that facilitates their social adaption, diminishes feelings of isolation, and enhances their ability to interact constructively with others (Meeus et al., 2005). Self-esteem, defined as the perception and valuation of oneself, is recognized to influence social behaviors by enhancing confidence and diminishing social fears, thereby promoting good interactions and aiding people in forging deeper relationships (Harter, 1990; Leary and Baumeister, 2000).

Despite the significance of these components, previous research often examines the effects of physical exercise on peer attachment and self-esteem in isolation, neglecting the potential interactions between these constructs within a more integrated framework. While previous studies have examined the individual effects of physical exercise on either peer attachment or self-esteem, few have explored the sequential pathway through which these psychological resources jointly mediate the relationship between physical activity and social adjustment. By addressing this gap, the present study offers a novel theoretical contribution through a sequential mediation model, integrating interpersonal and intrapersonal mechanisms in the context of university student development. This study gap signifies the need to investigate these constructs in conjunction, offering a more profound comprehension of how physical exercise may facilitate social adjustment by impacting peer affiliation and self-esteem.

Moreover, the university milieu in China offers a distinctive framework for analyzing these linkages; however, similar challenges in student adjustment have also been observed in other high-pressure academic contexts, such as in Korea and Japan (Ahn and Lee, 2016), suggesting that the benefits of physical exercise may extend across cultural boundaries. By situating our findings within these broader cultural comparisons, this study contributes to the international literature on how physical exercise can enhance student well-being across diverse educational systems. Chinese students often encounter significant academic expectations in a competitive educational setting, necessitating enhanced social adjustment to mitigate stress (Hu, 2019). Cultural influences, including the preponderance of single-child households, significantly affect the social dynamics and adaptability of pupils, since many may have increased challenges in adjusting to social changes owing to parental overprotection (Schaefer et al., 2011; Sun et al., 2019). Limited research has examined how physical exercise might function as a beneficial resource for these children, facilitating resilience, promoting independence, and aiding social adaptation in a challenging academic environment.

To sum up, this study investigates how physical exercise enhances social adjustment among Chinese university students through the sequential mediation of peer attachment and self-esteem. By integrating these variables into a unified model, the research clarifies the mechanisms linking physical activity and social adaptation, offering theoretical and practical insights into how universities might support students’ psychosocial development through exercise-based initiatives.

## 2 Literature review

### 2.1 Physical exercise and social adjustment

Physical exercise is widely acknowledged as a key component of personal health, enhancing not only physical fitness but also promoting cognitive, emotional, and social development. For university students—who are navigating a critical period of emerging adulthood marked by complex academic demands and evolving social roles—social adjustment becomes essential. Social adjustment refers to an individual’s capacity to navigate unfamiliar social environments, establish meaningful relationships, and respond effectively to interpersonal challenges (Zheng, 1999; Lin et al., 2024).

Physical exercise has been shown to strengthen psychological resilience and emotional regulation. Regular engagement in physical activity helps reduce anxiety, elevate mood, and improve goal-setting and self-control abilities (Holley et al., 2011; Sun et al., 2019). These cognitive and emotional capacities are particularly relevant for university students who frequently encounter academic pressure and transitional stress. Physical activity fosters a healthy emotional state, enabling students to approach academic and social demands with greater calmness, confidence, and adaptability (Sagatun et al., 2007).

The Broaden-and-Build Theory (Fredrickson, 2004), which highlights the role of positive emotions in expanding individuals’ thought-action repertoires, provides a theoretical foundation for the sequential mediation model proposed in this study. Physical exercise often elicits positive emotional states such as joy, contentment, and pride, which, according to the theory, facilitate the development of both interpersonal resources (e.g., peer attachment) and intrapersonal strengths (e.g., self-esteem). These resources, in turn, enhance students’ capacity to adapt to new social environments, thereby linking physical activity directly to social adjustment through a theoretically grounded mechanism.

While the benefits of physical exercise for emotional and social development are well-documented, some research suggests that these effects are not universal. For example, the extent to which physical activity enhances social adaptation may depend on contextual factors such as cultural norms, individual personality traits, or the quality of the exercise environment (Allender et al., 2006). In some cases, students who lack intrinsic motivation or experience negative peer dynamics during group exercise may not experience improvements in social functioning. These findings highlight the importance of considering moderators when evaluating the relationship between physical activity and social adjustment.

Beyond its emotional and cognitive effects, physical activity also serves as a platform for social connection. Participation in team sports, group fitness classes, or recreational physical activities provides structured opportunities for students to interact in supportive and collaborative environments. Such interactions foster communication skills, trust, cooperation, and a sense of belonging—key ingredients for effective social adjustment (Penedo and Dahn, 2005; Rose-Krasnor et al., 2006).

These social benefits are particularly pronounced in collectivist or high-pressure academic cultures, such as China’s. The limited availability of informal social interaction, combined with intense academic expectations, often restricts students’ opportunities to develop strong peer connections (Hu, 2019). In this context, organized physical exercise offers a rare and valuable space for peer bonding and emotional support. It fosters resilience and contributes to the development of social skills critical to student well-being and social integration (Liu and Qu, 2017).

### 2.2 The mediating role of peer attachment

Peer attachment denotes the emotional and social connections people establish with their peers, providing companionship, emotional support, and validation at critical life stages (Ainsworth, 1989; Allen, 1999). Within the university environment, where students often encounter heightened academic demands and the difficulties of social adaptation, peer attachment emerges as a critical determinant of social adjustment. Research indicates that robust peer connections provide a “safe haven” for students, enabling them to seek assistance, share experiences, and cope with stress, all of which are essential for their emotional stability and social development (Meeus et al., 2005; Parker and Asher, 1993). The function of peer bonding in facilitating social adaptation underscores its significance as a mediating element in the correlation between physical exercise and social adjustment.

The relationship between physical exercise and peer attachment may be comprehended via the social connections and collective experiences enabled by physical activities. Physical activity, especially in group contexts, offers a systematic and encouraging atmosphere for students to engage, converse, and cultivate trust (Liu and Qu, 2017). Participating in team sports, group exercises, or leisure activities enables students to cultivate connections with their classmates, establishing bonds that transcend the activity. As per the Social Development Theory (Vygotsky, 1978), learning and development transpire via social interactions; hence, physical exercise fosters optimal circumstances for students to participate in significant social exchanges, so reinforcing attachment relationships and enhancing social networks. Peer attachments, established via common objectives and reciprocal support, provide a fundamental component for students’ social adaptation within the larger university environment (Demanet and Van Houtte, 2012).

Moreover, peer connection offers pupils emotional stability, therefore improving their capacity to acclimate to novel social contexts. University students, particularly in the emerging adulthood stage, are more reliant on peer interactions for emotional and social support as they attain independence from their families (Allen and Land, 1999; Weiss, 1991). Engaging in physical activities that need collaboration, coordination, and collective effort fosters a feeling of connection and mutual understanding among students. This supporting network enhances their ability to confront social obstacles with increased confidence and resilience, directly influencing their capacity for social adjustment (Bagwell et al., 1998). Peer connection offers emotional validation that alleviates feelings of isolation, enhances a sense of closeness, and eventually augments students’ capacity to adjust to the challenges of university life.

Peer attachment promotes social support and facilitates the development of essential interpersonal skills for social adjustment. Consistent engagement in physical activities enables kids to develop and enhance communication, empathy, and problem-solving skills in a secure and organized setting. Attachment Theory (Bowlby, 1969) posits that stable attachments provide a foundation for the development of social competences by providing a dependable platform from which people may navigate social interactions and engage in various types of communication and cooperation. Through participation in physical activities with peers, children have the opportunity to refine their social skills, so improving their adaptability to various social environments and enhancing their overall social adjustment.

The significance of peer attachment as a mediator in this connection is further emphasized by cultural considerations. In China, where elevated academic standards and competitive settings may restrict students’ social relationships, the significance of peer attachment becomes more apparent. Research indicates that Chinese students often encounter increased stress from academic demands, and emotional support from peer relationships might mitigate these stresses, enhancing students’ coping mechanisms (Hu, 2019; Schaefer et al., 2011). Physical exercise fosters peer attachment, so acting as a significant resource for kids in high-pressure settings, facilitating the development of social networks that enhance their well-being and social integration (Liu and Qu, 2017; Sun et al., 2019).

### 2.3 The mediating role of self-esteem

Self-esteem, characterized as an individual’s comprehensive self-assessment or perception of self-worth, is a pivotal psychological element affecting social adaptation, particularly among university students facing intricate social and academic challenges (Leary and Baumeister, 2000; Rosenberg, 1965). Studies indicate that elevated self-esteem correlates with enhanced social confidence, resilience, and adaptability, facilitating improved social interactions and a more seamless adjustment to university environments (Harter, 1990; Ju et al., 2011). For adolescents transitioning to adulthood, self-esteem serves as an emotional foundation that improves their capacity to navigate social problems, manage interpersonal relationships, and assimilate into new social situations (Whitesell et al., 2009).

Physical activity significantly enhances self-esteem via several mechanisms. Consistent physical exercise enhances body image, fosters physical health, and elevates self-efficacy, all of which augment an individual’s self-worth and self-perception (Erkut and Tracy, 2002; Lin et al., 2024; Liu et al., 2015). University students who participate in physical exercise often express enhanced feelings of achievement and self-assurance resulting from their fitness advancements, thereby fostering a more robust and affirmative self-image. The enhancement of self-esteem subsequently promotes increased social engagement, as students with elevated self-regard are generally more at ease in initiating and maintaining social interactions, thereby better positioning them to acclimate to the social and academic milieu of university life (Thøgersen-Ntoumani et al., 2014).

The relationship among physical exercise, self-esteem, and social adjustment is elucidated by the Sociometer Theory (Leary and Baumeister, 2000; Leary et al., 1995), which asserts that self-esteem serves as an internal gauge of social belongingness. This theory posits that persons with elevated self-esteem see themselves as more socially acceptable and competent, hence motivating them to interact favorably with others and cultivate stronger social connections. Physical exercise enhances self-efficacy and body pleasure, so elevating students’ self-esteem, which in turn augments their social confidence and diminishes social worries (Haugen et al., 2011). This heightened self-esteem empowers students to engage in social circumstances with more assertiveness, promoting good interactions and efficient adaptation to new social networks and academic environments (Sun et al., 2019). Thus, self-esteem serves as a mediating variable that connects physical exercise to social adjustment by cultivating the social confidence essential for effective integration into university life.

Furthermore, self-esteem acts as a protective factor against stress and adverse self-image, so enhancing its function as an intermediary between physical exercise and social adaptation. University students sometimes face significant academic pressure, social comparison, and obstacles associated with independence, which may adversely affect their self-esteem. Physical exercise serves as a mechanism for alleviating stresses and enhancing mental well-being and resilience. Participating in physical activities may cultivate a feeling of accomplishment and proficiency, so bolstering students’ self-esteem and aiding them in maintaining a good self-image despite external constraints (Penedo and Dahn, 2005). As students’ self-esteem improves, they cultivate more emotional stability and coping mechanisms, enabling them to more effectively navigate the social and intellectual intricacies of university life (Allen, 1999).

The Broaden-and-Build Theory (Fredrickson, 2004) offers further theoretical justification for comprehending how physical exercise might indirectly enhance social adjustment by influencing self-esteem. This theory asserts that pleasant emotions expand people’s thought-action repertoires, facilitating the development of psychological and social resources that enhance long-term adaptation. Physical exercise often elicits pleasant feelings, including pleasure, contentment, and pride, which enhance pupils’ self-esteem and cultivate a favorable self-concept (Li et al., 2016). Enhanced self-esteem enables pupils to broaden their social relationships, facilitating the formation of new connections, participation in varied social experiences, and adaptation to novel surroundings. Consequently, by fostering good feelings and self-esteem, physical exercise indirectly improves adolescents’ social adjustment by augmenting their social resources and resilience.

Among Chinese university students, self-esteem serves as a significant mediator owing to the cultural prioritization of academic success and social cohesion. Elevated academic expectations may significantly impact students’ self-esteem, complicating their ability to navigate social and academic changes (Hu, 2019). Physical exercise serves as an effective means to mitigate these demands, enabling pupils to attain personal objectives outside academics, enhancing self-esteem and confidence. Physical exercise enhances self-esteem, enabling students to more successfully manage social and academic problems, and fostering a comprehensive approach to well-being and adaptation (Liu and Qu, 2017).

### 2.4 The sequential mediation model

Peer attachment and self-esteem interact considerably among university students, influencing their social adjustment. Peer attachment refers to the emotional connections established between people and their peers, providing a feeling of comfort, belonging, and emotional support (Ainsworth, 1989; Allen, 1999). For students entering the university setting, robust peer connections serve as a “social anchor,” offering a firm basis amidst heightened independence, academic obstacles, and social exploration (Meeus et al., 2005). As students cultivate these connections, they acquire not just a feeling of belonging but also affirmation from their peers, significantly impacting their self-esteem. Self-esteem is a crucial determinant of how people assess their value and proficiency in social contexts (Park and Park, 2015). When students see acceptance and support from their peers, they cultivate a more favorable self-image, resulting in elevated self-esteem (Pinheiro Mota and Matos, 2013; Whitesell et al., 2009).

The impact of peer connection on self-esteem may be elucidated by the Reflected Appraisals Theory, which asserts that self-esteem partially evolves from people’s perceptions of how others see them (Cooley, 1902). In university environments, where students increasingly depend on their peers for social validation and assistance, peer attachment emerges as a crucial determinant of self-esteem. Constructive peer connections provide validating feedback that enhances self-esteem and competence (Liu and Wei, 2008). When kids encounter robust, supportive relationships, they assimilate these positive perspectives, resulting in enhanced self-esteem. In contrast, insecure or fragile peer bonds may result in feelings of social isolation, reduced self-esteem, and heightened vulnerability to social anxiety and self-doubt (Thompson et al., 2016). The association between peer attachment and self-esteem highlights the need of cultivating healthy peer interactions to develop a solid self-concept and improve psychological resilience.

Moreover, peer connection fosters a feeling of social belonging and enhances students’ capacity to manage stress and confront obstacles with a positive outlook, all of which are crucial for sustaining high self-esteem. Attachment Theory (Bowlby, 1969) posits that stable bonds provide a “safe base” enabling people to explore their surroundings with assurance. University students, confronted with many academic and social demands, get their secure basis from helpful peer interactions. When adolescents experience safe peer bonds, they are more inclined to engage in new social settings with confidence, therefore reinforcing favorable self-evaluations. These safe relationships cultivate an atmosphere in which students feel valued, respected, and understood, all of which are essential to a robust sense of self-esteem (Laible et al., 2004; Millings et al., 2012). Thus, peer connection serves as a safeguard against social stresses, preserving self-esteem from variations that may result from academic demands or social difficulties.

In the sequential mediation concept, physical exercise acts as a catalyst that opens a route toward improved peer bonding, which then enhances self-esteem and eventually promotes social adjustment. Physical exercise, especially in group or team contexts, facilitates social interaction and bonding, enabling pupils to establish close peer connections (Lim and Lee, 2017; Rose-Krasnor et al., 2006). These situations promote collaboration, cooperation, and common objectives, hence enhancing peer connections. Consistent engagement in physical activities fosters friendships and cultivates a feeling of camaraderie and mutual support that strengthens peer relationships. As these ties strengthen, kids get emotional comfort and affirmation from their peers, which directly influences their self-esteem (Schaefer et al., 2011). Students engaged in team sports may gain support and recognition from their peers, so enhancing their self-esteem and cultivating a good self-image.

The augmentation of self-esteem via peer affiliation, promoted by physical activity, is elucidated by the Sociometer Theory (Leary and Baumeister, 2000). This idea posits that self-esteem serves as an internal measure of social acceptability and belongingness. When kids experience connection and acceptance within their peer group, as seen in physical exercise settings, their self-esteem is favorably reinforced. This social acceptance results in enhanced confidence during social interactions, alleviating social anxiety and promoting improved adaptation to unfamiliar social settings. Consequently, students with heightened self-esteem are more adept at managing the intricacies of university social life, interacting meaningfully with peers, and surmounting interpersonal difficulties, which are essential components of social adjustment (Haugen et al., 2011; Pinto et al., 2015).

The Broaden-and-Build Theory (Fredrickson, 2004) provides more understanding of how physical training indirectly improves social adjustment via this mechanism. This idea asserts that pleasant emotions, such as those elicited by physical exercise, expand an individual’s consciousness and foster the cultivation of enduring social and psychological resources. Physical exercise often elicits positive emotions, including pleasure, contentment, and pride, which enable children to engage in social settings with an open and confident disposition. These pleasant emotions strengthen peer connections by cultivating trust and mutual respect in encounters, therefore reinforcing self-esteem. Elevated self-esteem enables students to effectively manage social and intellectual obstacles, resulting in enhanced social adjustment as they traverse the university landscape with resilience and confidence.

**Figure 1:**
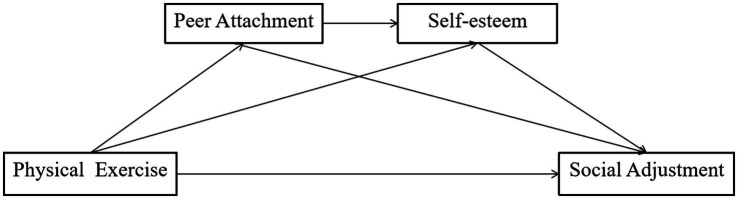
The hypothetical chain mediating effect model.

This sequential mediation model posits that social adjustment is the final result shaped by the cumulative impacts of physical activity, peer attachment, and self-esteem. The connection between physical exercise and social adjustment via peer attachment and self-esteem illustrates that physical activity promotes not only physical health but also social and psychological growth. Physical exercise facilitates peer connections among students, enhancing self-esteem and equipping them to navigate new social contexts with confidence and flexibility (Thøgersen-Ntoumani et al., 2014). This model exemplifies a comprehensive approach to student well-being, integrating physical, social, and psychological elements to enhance favorable social adjustment results. The theories are delineated as follows (refer to Figure 1).

*H1*: Physical exercise positively impacts social adjustment among university students.

*H2*: Physical exercise indirectly influences social adjustment through peer attachment.

*H3*: Physical exercise indirectly influences social adjustment through self-esteem.

*H4*: Physical exercise has a sequential indirect effect on social adjustment through both peer attachment and self-esteem.

## 3 Methods

### 3.1 Participants and procedure

This research included 672 university students (432 males and 240 females) aged 18–22 (Mage = 20.37, SD = 1.14), participating in several majors at a university in Southwest China. The sample size was determined based on recommendations for adequate power in mediation analysis and included students across all academic years to capture a broad range of university adjustment experiences. Participants were included if they were full-time students and had no physical conditions restricting regular exercise participation. The sample included individuals from various academic levels: 56% were freshmen, 27.1% were sophomores, 9.2% were juniors, and 7.7% were seniors. The university’s ethics committee authorized the research. Collaboration was obtained from student counselors who enabled data collection access. All participants given written informed permission, were guaranteed anonymity, and were advised of their right to withdraw at any moment without consequence. The data collection occurred over a duration of 4 weeks. Participants conducted the survey using the Wenjuanxing platform. The survey lasted around 15 min, and participants were compensated with a nominal sum of RMB 10 for their participation.

### 3.2 Measures

#### 3.2.1 Physical exercise

Physical exercise was assessed via a single-item scale modified from Sagatun et al. (2007), asking participants, “How many hours per week do you partake in physical activities that induce sweating or heavy breathing?” Responses were coded from 0 to 5. While this measure has demonstrated acceptable validity in previous adolescent studies, its reliance on self-report and ordinal scaling introduces potential measurement limitations. Future research should consider using validated instruments such as the International Physical Activity Questionnaire (IPAQ) or objective tools like accelerometers to improve reliability and accuracy.

#### 3.2.2 Peer attachment

The evaluation of peer attachment used a modified version of the Inventory of Parental and Peer Attachment (IPPA-R), updated by Kong (2017) for college students, which measures cognitive views and emotions about peers across three dimensions: trust, communication, and alienation. This 24-item scale has statements such as “I can depend on friends to assist me in managing emotional challenges” and “I experience loneliness or detachment in the company of my friends” (reverse-coded). Responses were evaluated using a five-point Likert scale (1 = strongly disagree to 5 = strongly agree), where higher scores indicate more peer affiliation. This measure has strong reliability and validity among Chinese university students, shown by a Cronbach’s alpha of 0.85.

#### 3.2.3 Self-esteem

Self-esteem was assessed with the Rosenberg Self-Esteem Scale (Rosenberg, 1965), a well-validated 10-item self-report instrument for evaluating overall self-worth. Items were evaluated using a four-point Likert scale, from 1 (very much so) to 4 (not at all). The scale comprises comments like “I often feel I am worthless” and “I am content with myself,” with the majority of questions reverse-scored to get a total score between 10 and 40, where elevated values indicate more self-esteem. Our measure has robust reliability, shown by a Cronbach’s alpha of 0.82 in our sample, and has been extensively used among adolescents and young adults across many cultural settings (Schmitt and Allik, 2005).

#### 3.2.4 Social adjustment

The Social Adjustment Scale, created by Zheng in 1999, was used to evaluate social adjustment, including 20 questions that examine three subdimensions: learning adjustment, interpersonal adjustment, and life adjustment. Examples of items are “I am eager to acquire new knowledge and subjects as they motivate me” (learning adjustment), “I can effortlessly interact with others in a novel environment” (interpersonal adjustment), and “I can adapt irrespective of alterations in my living conditions” (life adjustment). Odd-numbered things are rated from 1 to 5 (with 1 representing “Yes,” 3 indicating “Uncertain,” and 5 denoting “No”), but even-numbered items are evaluated in reverse order. Elevated scores indicate enhanced social adjustment. This scale has strong internal consistency, shown by a Cronbach’s alpha of 0.89, and demonstrates good construct validity in prior research (Liao et al., 2016).

### 3.3 Statistical analysis

All statistical analyses were conducted using SPSS version 27.0. Initially, descriptive statistics and Pearson’s correlation coefficients were computed for all principal variables: physical exercise, peer attachment, self-esteem, and social adjustment. These correlations provide a preliminary comprehension of the connections among variables. The suggested mediation model was analyzed using Hayes (2013) PROCESS macro for SPSS, especially Model 6, which evaluates sequential mediation. In this model, physical exercise was considered the independent variable, social adjustment the dependent variable, and peer attachment and self-esteem as sequential mediators. The research used a bootstrapping method with 5,000 resamples to determine the indirect effects, and the mediation effect was considered significant if the 95% confidence interval excluded zero.

## 4 Results

### 4.1 Common method Bias

Due to the collection of all data via self-report questionnaires, a Harman’s single-factor test was performed to assess potential common method bias, with the first factor accounting for 14.35% of the variance—below the 40% threshold. However, we acknowledge that this test is limited in its sensitivity. Future studies are encouraged to adopt more rigorous procedures, such as latent method factor modeling or procedural remedies like temporal separation, to better control for common method variance.

### 4.2 Descriptive statistics and correlation analysis of each variable

Table 1 presents the means, standard deviations, and correlations for physical exercise, peer attachment, self-esteem, and social adjustment. Results revealed that physical exercise was positively correlated with social adjustment (*r* = 0.39, *p* < 0.01), peer attachment (*r* = 0.13, *p* < 0.01), and self-esteem (*r* = 0.53, *p* < 0.01). Similarly, peer attachment was significantly correlated with both social adjustment (*r* = 0.33, *p* < 0.01) and self-esteem (*r* = 0.18, *p* < 0.01). Self-esteem also demonstrated a strong positive correlation with social adjustment (*r* = 0.53, *p* < 0.01).

**Table 1:** Descriptive statistical results of variables and Pearson correlations (*N* = 672).

Variables	Mean	SD	1	2	3	4
1. Social Adjustment	48.65	18.18	1			
2. Physical Exercise	2.15	0.78	0.39^***^	1		
3. Peer Attachment	35.80	17.00	0.33^***^	0.13^**^	1	
4. Self-esteem	18.30	6.70	0.53^***^	0.53^***^	0.18^***^	1

**P < 0.01, ***P < 0.001.

### 4.3 Testing of the chain mediation effect of peer attachment and self-esteem

The SPSS macro process program developed by Hayes was used to examine the mediating influence of peer attachment and self-esteem on the link between physical exercise and social adjustment. The research accounted for sex and age variables. However, we acknowledge that other potentially important control variables—such as socio-economic status and prior sports participation—were not included in the analysis. Future research should incorporate a broader range of covariates to control for confounding influences on the observed associations. Process Model 6 of v3.4 was implemented, in detail (refer to Tables 2, 3).

**Table 2:** Regression analysis of variable relationship in chain intermediary model.

Regression equation		Overall fitting index			Significance of regression coefficient		
Result variables	Predictive variable	R	R^2^	*F*	β	*t*	*P*
Peer Attachment	Physical exercise	0.16	0.03	6.77^**^	0.028	4.38**	0.0011
Self-esteem	Physical exercise	0.54	0.31	71.68^***^	0.504	17.12***	< 0.001
	Peer attachment				0.473	4.51***	0.0007
Social adjustment	Physical exercise	0.61	0.36	76.92^***^	0.102	4.96***	0.0001
	Peer attachment				0.706	8.23***	< 0.001
	Self-esteem				0.305	12.62***	< 0.001

**P < 0.01, ***P < 0.001.

**Table 3:** Analysis of mediating effect between peer attachment and self-esteem.

Indirect effect	Effect value	Boot SE	95% CI		Relative mediating effect
			LLCI	ULCI	
Physical Exercise → Peer Attachment → Social Adjustment (indirect1)	0.0376	0.0101	0.0091	0.0588	8.00%
Physical Exercise → self-esteem → Social Adjustment (indirect2)	0.2101	0.0249	0.2730	0.3690	56.50%
Physical Exercise → Peer Attachment → Self-esteem → Social Adjustment (indirect3)	0.0055	0.0026	0.0023	0.0224	2.54%
Total Indirect Effect	0.2532	0.0269	0.1999	0.2944	67.07%

The significance of the above mediation effect was tested by using the PROCESS program and bootstrap method. According to the hypothesis model, physical exercise is associated with social adjustment through four paths. Direct path: physical exercise → social adjustment; indirect path 1: physical exercise → peer attachment → social adjustment; indirect path 2: physical exercise → self-esteem → social adjustment; indirect path 3: physical exercise → peer attachment → self-esteem → social adjustment. If any of the three indirect paths were significant, this suggested mediation.

**Figure 2:**
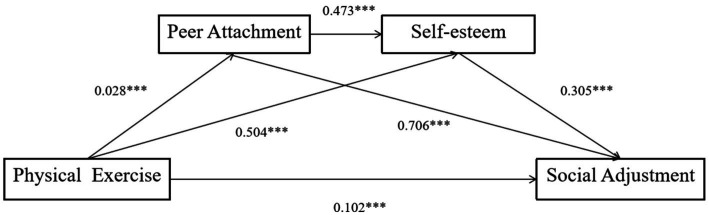
Results of the final chain mediation model. ***p < 0.001.

As shown in Table 3 and Figure 2, the indirect path 1 effect value was 0.0376 (95% CI: 0.0091–0.0588), the indirect path 2 effect value was 0.2101 (95% CI: 0.2730–0.3690), and the indirect path 3 effect value was 0.0055 (95% CI: 0.0023–0.0224). While all three indirect effects were statistically significant, the sequential effect (indirect3) was small in magnitude, suggesting limited practical significance. Nonetheless, it provides preliminary evidence for a theoretically meaningful pathway that warrants further exploration. The mediating effect ratio was 67.07%, indicating a substantial proportion of the total effect is mediated through peer attachment and self-esteem. The hypothesis model of this study was supported by the data analysis results.

The regression model demonstrated acceptable explanatory power. For example, the final model predicting social adjustment yielded an R^2^ of 0.36, indicating that approximately 36% of the variance in social adjustment could be explained by physical exercise, peer attachment, and self-esteem. This contextualizes the model’s strength and offers insight into its predictive capacity. While η^2^ was not directly computed, these R^2^ values provide a reasonable approximation of effect size.

## 5 Discussion

This research investigates the correlation between physical exercise and social adjustment in Chinese university students, focusing on the sequential mediating roles of peer attachment and self-esteem. This research examines mediators to elucidate how physical exercise fosters not just physical health but also social and psychological growth, hence improving students’ social adaptation within the university environment. However, given the cross-sectional nature of the data, the findings do not imply causation. Therefore, caution is warranted in interpreting the observed relationships as causal.

### 5.1 General findings

Hypothesis 1 received empirical support. This discovery corresponds with studies indicating that physical exercise furnishes a structured environment for university students, particularly those undergoing transitional life phases, by facilitating social contact and collaboration. Engaging in physical activities may substantially enhance social skills and facilitate adaptation for students adjusting to new academic and social environments (Schaefer et al., 2011); however, these benefits may not be universal. Individual differences such as gender, cultural orientation, prior athletic background, and baseline social competence may moderate these effects and should be considered in future research. Physical exercise often has cooperative components such as team sports, which intrinsically promote teamwork, common objectives, and communication. These factors may assist students in improving their interpersonal skills, a crucial factor for effective social integration in a university environment. Moreover, prior research suggests that physical exercise enhances psychological resilience and self-regulation, which are essential for students as they confront the obstacles of higher education, social identity transitions, and heightened independence (Penedo and Dahn, 2005). Through participation in physical activities, students develop self-discipline and stress management, essential abilities for balancing academic obligations and social integration.

Hypothesis 2 received support, demonstrating that peer connection serves a crucial mediation function in the correlation between physical exercise and social adjustment. University students, particularly throughout the emerging adulthood stage, often start to depend more on their friends than on their relatives. This transition establishes peer attachment as a key support system that satisfies emotional and social needs (Meeus et al., 2005). Physical activity, especially in group or team environments, creates an optimal background for promoting peer connections. Through these activities, kids develop trust, collaboration, and camaraderie with others, establishing significant connections. Peer relationships provide emotional support and a feeling of belonging, essential for adjusting to social changes (Allen and Land, 1999; Demanet and Van Houtte, 2012; Liu and Qu, 2017). Supportive peer interactions serve as a buffer against isolation and assist in maintaining psychological stability for kids experiencing academic stress and social changes. Consequently, physical exercise plays a crucial role in fostering peer connections, which is advantageous for both social bonding and the improvement of emotional resilience.

Hypothesis 3 was also validated, highlighting the significance of self-esteem as a mediating variable. Physical exercise enhances self-esteem via boosting physical self-image and fostering a feeling of achievement. University students who participate in physical activities may experience improved fitness and health awareness, hence boosting body satisfaction and self-esteem (Liu et al., 2015). Enhancements in self-esteem provide a heightened feeling of confidence in social contexts, enabling pupils to engage in conversations with a positive self-image. This, therefore, enhances their social adaptation, since kids with elevated self-esteem are often more resilient and adept at managing both social and academic obstacles (Leary and Baumeister, 2000). Elevated self-esteem enhances students’ self-perception and influences their interactions with others, often resulting in more rewarding and affirmative social exchanges. Consequently, physical exercise acts as a conduit for personal empowerment, establishing a basis for improved social adaptation via elevated self-perception and resilience.

Hypothesis 4 was validated, demonstrating that physical exercise promotes social adjustment via a sequential mediation effect that encompasses both peer attachment and self-esteem. This route highlights the dual influence of physical exercise on interpersonal and intrapersonal development. Exercise enables pupils to reinforce peer relationships, offering the social support essential for boosting self-esteem. When students perceive more support and worth from their peers, their self-perception is enhanced, resulting in heightened confidence and flexibility in social contexts (Whitesell et al., 2009). The sequential mediation effect underscores a cumulative association, whereby physical exercise is linked to stronger peer connections, which may enhance self-esteem and, in turn, are associated with better social adjustment outcomes. This discovery endorses a comprehensive perspective on social adaptation, whereby social and psychological elements collaboratively facilitate the growth and adjustment of university students. This route demonstrates how physical exercise has a synergistic impact, cultivating a feeling of community and self-worth that improves general well-being and social adaptation (Laible et al., 2004).

### 5.2 Implications

This work provides a substantial theoretical addition by developing a sequential mediation model that clarifies the indirect mechanisms by which physical exercise (PE) enhances social adjustment in university students, mediated by peer attachment and self-esteem. Physical exercise is generally recognized as advantageous for social and psychological well-being, functional capacity, and overall quality of life (Allender et al., 2006), and has been shown to diminish the risk of coronary heart disease and certain malignancies. Previous studies have highlighted the significance of these variables—physical education’s impact on self-esteem (Liu et al., 2015) and peer attachment’s influence on social adaption (Schaefer et al., 2011); yet, this research synthesizes these concepts into a unified framework. The research confirms that physical education favorably affects social adjustment by improving peer attachment and self-esteem, emphasizing the intricate relationship between social and psychological elements in the adaptation processes of young people (Penedo and Dahn, 2005; Whitesell et al., 2009). This integrated approach enhances the current research on adolescent social development by illustrating how physical exercise fosters conditions that promote interpersonal interaction, hence strengthening self-esteem and social resilience (Allen and Land, 1999). Furthermore, the results enhance the comprehension of physical education’s influence beyond physical health, establishing it as a vital element in the mental well-being and social adaptability of university students. This study endorses a biopsychosocial model of development, illustrating the interconnection of physical, social, and psychological factors in enhancing social functioning throughout transitional life phases (Laible et al., 2004).

This research highlights the need of integrating regular physical exercise programs inside university environments to improve students’ social adaptation. Universities may foster supportive peer connections among students by providing team sports, group exercise sessions, and fitness programs, which, as shown by this research, enhance social adjustment (Liu and Qu, 2017; Schaefer et al., 2011). Practitioners and educators must emphasize the creation of accessible physical education opportunities, since they enhance physical health and serve as a conduit for cultivating social and emotional resilience (Thøgersen-Ntoumani et al., 2014). Moreover, initiatives designed to strengthen peer bonding and self-esteem may improve the efficacy of physical education programs in facilitating social adjustment. Initiatives that promote collaborative objectives, peer engagement, and self-efficacy activities may optimize the psychological and social advantages of physical exercise. Consistent with the findings of the present study, focused initiatives to enhance peer relationships and self-image may yield comprehensive strategies for student well-being, tackling the varied challenges encountered by university students during their academic, social, and personal transitions (Armsden and Greenberg, 1987; Leary and Baumeister, 2000; Sun et al., 2019). Nonetheless, it is crucial to recognize that the effects of such initiatives may vary based on individual characteristics. Tailored interventions that consider gender, personal history with physical activity, and existing social skill levels may enhance the effectiveness of physical exercise-based programs. It is also important to recognize that physical exercise encompasses diverse forms, ranging from structured team sports to solitary activities. These different modalities may offer varying degrees of social interaction and psychological benefits. For instance, team sports may more strongly promote peer bonding, whereas individual exercises may be more effective in building personal self-discipline and internal resilience. Future research should consider disaggregating the types of exercise to examine their distinct contributions to social adjustment.

### 5.3 Limitations

This research, while providing useful insights, has some limitations that must be recognized. The cross-sectional design restricts the capacity to draw causal implications about the links among physical exercise, peer attachment, self-esteem, and social adjustment. Importantly, as the study employed a cross-sectional design, it cannot establish causal relationships between variables. While the findings indicate significant associations, longitudinal or experimental research is needed to determine causal directions.

Secondly, the dependence on self-reported data adds possible biases, including social desirability and recollection bias, which may compromise the accuracy of participants’ replies about their physical activity levels, peer connections, and self-esteem. Employing objective metrics, such as fitness monitors for physical activity or independent assessments for social adaptation, may improve data dependability and mitigate subjective biases.

Thirdly, the study only controlled for gender and age, while omitting other relevant background variables such as socio-economic status and prior physical activity experience. These unmeasured variables may act as confounders and limit the generalizability of the findings. Future research should include a broader array of control variables to better isolate the effects of physical exercise on social adjustment.

The research sample was sourced from a single institution in Southwest China, which may limit the generalizability of the findings. Differences in educational systems, rural versus urban settings, and cultural contexts (e.g., international populations) may yield different patterns of association and should be considered in future research.

The research sample was sourced from a single institution in Southwest China, perhaps limiting the generalizability of the results. Cultural and institutional variables specific to this environment may affect the observed connections; thus, future research should include samples from varied cultural and educational backgrounds to corroborate these results and investigate potential cross-cultural differences.

Additionally, this research failed to include possible moderating and mediating factors such as personality traits (e.g., resilience, self-efficacy), family support, and academic stress, all of which may interact with physical exercise to influence social adjustment. These variables could independently or jointly affect peer attachment and self-esteem, thereby altering the pathway proposed in the current model. Future research should consider incorporating such factors to achieve a more comprehensive understanding of the mechanisms underlying social adaptation. Integrating these variables in further study may provide a more refined understanding of how individual and contextual factors influence these dynamics among university students. Additionally, external influences such as family environment, socio-economic status, and broader cultural norms were not measured in this study. These factors could potentially affect the relationships among physical exercise, peer attachment, self-esteem, and social adjustment and should be considered in future research.

Finally, this study did not distinguish between different forms of physical exercise, such as team-based versus individual or structured versus unstructured activities. These variations may influence the degree and nature of psychosocial outcomes, and future research should explore these distinctions to refine intervention strategies.

## 6 Conclusion

This research emphasizes the significance of physical exercise in being associated with better social adjustment among university students through the sequential mediation of peer attachment and self-esteem. This sequential mediation model highlights how physical activity is associated with improvements in students’ social and emotional resilience by interacting with psychological mechanisms. However, due to the cross-sectional nature of the study and reliance on self-reported measures, conclusions should be interpreted with caution. Future research should adopt longitudinal designs and incorporate objective measures to validate these pathways. Additionally, given the homogeneous nature of the sample (students from a single institution in Southwest China), the external validity of the findings is limited. Future research should replicate the model across diverse cultural and educational settings and consider testing alternative pathways, such as parallel mediation, to further examine the robustness of the proposed mechanisms.

## Data Availability

The original contributions presented in the study are included in the article/supplementary material, further inquiries can be directed to the corresponding author.

## References

[ref1] Ahn J. A. Lee S. (2016). Peer attachment, perceived parenting style, self-concept, and school adjustments in adolescents with chronic illness. Asian Nurs. Res. 10, 300–304. doi: 10.1016/j.anr.2016.10.003, PMID: 28057318

[ref2] Ainsworth M. D. S. (1989). Attachment beyond infancy. Am. Psychol. 44, 709–716. doi: 10.1037/0003-066X.44.4.709, PMID: 2729745

[ref3] Allen J. P. (1999). Handbook of attachment: Theory, research, and clinical applications. New York: Guilford Press.

[ref4] Allen J. P. Land D. (1999). “Attachment in adolescence” in Handbook of attachment: Theory, research, and clinical applications. eds. Cassidy J. Shaver P. R. (New York: Guilford Press), 319–335.

[ref5] Allender S. Cowburn G. Foster C. (2006). Understanding participation in sport and physical activity among children and adults: a review of qualitative studies. Health Educ. Res. 21, 826–835. doi: 10.1093/her/cyl063, PMID: 16857780

[ref6] Armsden G. C. Greenberg M. T. (1987). The inventory of parent and peer attachment: individual differences and their relationship to psychological well-being in adolescence. J. Youth Adolesc. 16, 427–454. doi: 10.1007/BF02202939, PMID: 24277469

[ref7] Arnett J. J. (2000). Emerging adulthood: a theory of development from the late teens through the twenties. Am. Psychol. 55, 469–480. doi: 10.1037/0003-066X.55.5.469, PMID: 10842426

[ref8] Bagwell C. L. Newcomb A. F. Bukowski W. M. (1998). Preadolescent friendship and peer rejection as predictors of adult adjustment. Child Dev. 69, 140–153. doi: 10.1111/j.1467-8624.1998.tb06139.x, PMID: 9499563

[ref9] Bowlby J. (1969). Attachment and loss: Attachment. Basic Books.

[ref10] Chisholm L. Hurrelmann K. (1995). Adolescence in modern Europe. Pluralized transition patterns and their implications for personal and social risks. J. Adolesc. 18, 129–158. doi: 10.1006/jado.1995.1010

[ref11] Cooley C. H. (1902). The looking-glass self. Prod. Reality 6, 126–128.

[ref12] Demanet J. Van Houtte M. (2012). School belonging and school misconduct: the differing role of teacher and peer attachment. J. Youth Adolesc. 41, 499–514. doi: 10.1007/s10964-011-9674-2, PMID: 21567214

[ref13] Erkut S. Tracy A. J. (2002). Predicting adolescent self-esteem from participation in school sports among Latino subgroups. Hisp. J. Behav. Sci. 24, 409–429. doi: 10.1177/0739986302238212, PMID: 21379403 PMC3048356

[ref14] Fredrickson B. L. (2004). The broaden-and-build theory of positive emotions. Philos. Trans. R. Soc. B Biol. Sci. 359, 1367–1377. doi: 10.1098/rstb.2004.1512, PMID: 15347528 PMC1693418

[ref15] Harter S. (1990). “Self and identity development” in At the threshold: The developing adolescent. eds. Feldman S. S. Elliott G. R. (Cambridge, MA: Harvard University Press), 352–387.

[ref16] Haugen T. Säfvenbom R. Ommundsen Y. (2011). Physical activity and global self-worth: the role of physical self-esteem indices and gender. Ment. Health Phys. Act. 4, 49–56. doi: 10.1016/j.mhpa.2011.07.001

[ref17] Hayes A. F. (2013). Introduction to mediation, moderation, and conditional process analysis: A regression-based approach. New York: Guilford Press.

[ref18] Holley J. Crone D. Tyson P. Lovell G. (2011). The effects of physical activity on psychological well-being for those with schizophrenia: a systematic review. Br. J. Clin. Psychol. 50, 84–105. doi: 10.1348/014466510X496220, PMID: 21332522

[ref19] Hu Q. (2019). The effect of increased intensity of physical exercises on mental health and resilience among college students. Chin. J. School Health 40, 89–91.

[ref20] Ju X. Liu X. Fang X. (2011). Research on adolescent parents and peer attachement in relation to self-esteem and social adaptation. Psychol. Dev. Educ. 12, 174–180.

[ref21] Kong R. (2017). Reliability and validity test of the revised parent peer dependency scale (IPPA-R). J. Shanxi Datong Univ. 33, 85–88. doi: 10.1007/s40299-022-00678-x

[ref22] Laible D. J. Carlo G. Roesch S. C. (2004). Pathways to self-esteem in late adolescence: the role of parent and peer attachment, empathy, and social behaviours. J. Adolesc. 27, 703–716. doi: 10.1016/j.adolescence.2004.05.005, PMID: 15561312

[ref23] Leary M. R. Baumeister R. F. (2000). The nature and function of self-esteem: sociometer theory. Adv. Exp. Soc. Psychol. 32, 1–62. doi: 10.1016/S0065-2601(00)80003-9

[ref24] Leary M. R. Tambor E. S. Terdal S. K. Downs D. L. (1995). Self-esteem as an interpersonal monitor: the sociometer hypothesis. J. Pers. Soc. Psychol. 68, 518–530. doi: 10.1037/0022-3514.68.3.518

[ref25] Li R. Bunke S. Psouni E. (2016). Attachment relationships and physical activity in adolescents: the mediation role of physical self-concept. Psychol. Sport Exerc. 22, 160–169. doi: 10.1016/j.psychsport.2015.07.003

[ref26] Liao H. Y. Zhong Y. H. Wang R. R. Tang H. (2016). Mobile phone addiction, self-esteem, shyness and interpersonal communication disturbance. Chin. J. Clin. Psych. 24, 852–855.

[ref27] Lim Y. Lee O. (2017). Relationships between parental maltreatment and adolescents’ school adjustment: mediating roles of self-esteem and peer attachment. J. Child Fam. Stud. 26, 393–404. doi: 10.1007/s10826-016-0573-8

[ref28] Lin H. Wang B. Hu Y. Song X. Zhang D. (2024). Physical activity and interpersonal adaptation in Chinese adolescents after COVID-19: the mediating roles of self-esteem and psychological resilience. Psychol. Rep. 127, 1156–1174. doi: 10.1177/00332941221137233, PMID: 36314269 PMC9618912

[ref29] Liu Y. Qu K. (2017). Research on the relationship between college students’ participation in sports and interpersonal adaptation. High. Educ. Explorat. 5, 113–116.

[ref30] Liu C. Wei Y. (2008). Research on middle school student’s self-esteem and interpersonal relationship development. Continue Educ. Res. 9, 103–105.

[ref31] Liu M. Wu L. Ming Q. (2015). How does physical activity intervention improve self-esteem and self-concept in children and adolescents? Evidence from a meta-analysis. PLoS One 10:e0134804. doi: 10.1371/journal.pone.0134804, PMID: 26241879 PMC4524727

[ref32] Meeus W. Iedema J. Maassen G. Engels R. (2005). Separation-individuation revisited: on the interplay of parent–adolescent relations, identity and emotional adjustment in adolescence. J. Adolesc. 28, 89–106. doi: 10.1016/j.adolescence.2004.07.003, PMID: 15683637

[ref33] Millings A. Buck R. Montgomery A. Spears M. Stallard P. (2012). School connectedness, peer attachment, and self-esteem as predictors of adolescent depression. J. Adolesc. 35, 1061–1067. doi: 10.1016/j.adolescence.2012.02.015, PMID: 22460237

[ref34] Park K. M. Park H. (2015). Effects of self-esteem improvement program on self-esteem and peer attachment in elementary school children with observed problematic behaviors. Asian Nurs. Res. 9, 53–59. doi: 10.1016/j.anr.2014.11.003, PMID: 25829211

[ref35] Parker J. G. Asher S. R. (1993). Friendship and friendship quality in middle childhood: links with peer group acceptance and feelings of loneliness and social dissatisfaction. Dev. Psychol. 29, 611–621. doi: 10.1037/0012-1649.29.4.611

[ref36] Penedo F. J. Dahn J. R. (2005). Exercise and well-being: a review of mental and physical health benefits associated with physical activity. Curr. Opin. Psychiatry 18, 189–193. doi: 10.1097/00001504-200503000-00013, PMID: 16639173

[ref37] Pinheiro Mota C. Matos P. M. (2013). Peer attachment, coping, and self-esteem in institutionalized adolescents: the mediating role of social skills. Eur. J. Psychol. Educ. 28, 87–100. doi: 10.1007/s10212-012-0103-z

[ref38] Pinto A. Veríssimo M. Gatinho A. Santos A. J. Vaughn B. E. (2015). Direct and indirect relations between parent–child attachments, peer acceptance, and self-esteem for preschool children. Attach Hum. Dev. 17, 586–598. doi: 10.1080/14616734.2015.1093009, PMID: 26426975

[ref39] Rose-Krasnor L. Busseri M. A. Willoughby T. Chalmers H. (2006). Breadth and intensity of youth activity involvement as contexts for positive development. J. Youth Adolesc. 35, 365–379. doi: 10.1007/s10964-006-9037-6 17087563

[ref40] Rosenberg M. (1965). Society and the adolescent self-image. Princeton, NJ: Princeton University Press.

[ref41] Sagatun Å. (2010). Physical activity and mental health in adolescence – A longitudinal study in a multiethnic cohort. Norway: University of Oslo.

[ref42] Sagatun A. Søgaard A. J. Bjertness E. Selmer R. Heyerdahl S. (2007). The association between weekly hours of physical activity and mental health: a three-year follow-up study of 15–16-year-old students in the city of Oslo, Norway. BMC Public Health 7, 1–9. doi: 10.1186/1471-2458-7-155, PMID: 17626617 PMC1955440

[ref43] Schaefer D. R. Simpkins S. D. Vest A. E. Price C. D. (2011). The contribution of extracurricular activities to adolescent friendships: new insights through social network analysis. Dev. Psychol. 47, 1141–1152. doi: 10.1037/a0024091, PMID: 21639618 PMC3134619

[ref44] Schmitt D. P. Allik J. (2005). Simultaneous administration of the Rosenberg self-esteem scale in 53 nations: exploring the universal and culture-specific features of global self-esteem. J. Pers. Soc. Psychol. 89, 623–642. doi: 10.1037/0022-3514.89.4.623, PMID: 16287423

[ref45] Sun S. Liu B. Sun Y. Chen P. Guo Z. (2019). An empirical study on the relationship between youth sports participation and social adjustment: taking tsinghua university as a case. J. Beijing Sport Univ. 42, 81–900.

[ref46] Thøgersen-Ntoumani C. Loughren E. A. Taylor I. M. Duda J. L. Fox K. R. (2014). A step in the right direction? Change in mental well-being and self-reported work performance among physically inactive university employees during a walking intervention. Ment. Health Phys. Act. 7, 89–94. doi: 10.1016/j.mhpa.2014.06.004

[ref47] Thompson H. M. Wojciak A. S. Cooley M. E. (2016). Self-esteem: a mediator between peer relationships and behaviors of adolescents in foster care. Child Youth Serv. Rev. 66, 109–116. doi: 10.1016/j.childyouth.2016.05.003

[ref48] Vygotsky L. (1978). Mind in society: The development of higher psychological processes. Harvard: Harvard University Press.

[ref49] Weiss R. S. (1991). “The attachment bond in childhood and adulthood” in Attachment across the life cycle. eds. Parkes C. M. Stevenson-Hinde J. Marris P. (London: Routledge), 66–76.

[ref50] Whitesell N. R. Mitchell C. M. Spicer P. (2009). A longitudinal study of self-esteem, cultural identity, and academic success among American Indian adolescents. Cult. Divers. Ethn. Minor. Psychol. 15, 38–50. doi: 10.1037/a0013456, PMID: 19209979 PMC2678750

[ref51] Xiong M. Guo X. Zhou Z. (2011). Effects of exercise behavior, experience, and willingness on college students’ psychology health. J. Wuhan Instit. Phys. Educ. 45, 48–51.

[ref52] Yan J. Tao B. Shi L. Lou H. Li H. Liu M. (2020). The relationship between extracurricular physical exercise and school adaptation of adolescents: a chain mediating model and gender difference. China Sport Sci. Technol. 56, 11–18.

[ref53] Yolanda B. M. Jesus S. G. Pedro F. G. J. Daniel C. M. Santos V. (2021). Effects of high-intensity interval training and moderate-intensity training on stress, depression, anxiety, and resilience in healthy adults during coronavirus disease 2019 confinement: a randomized controlled trial. Front. Psychol. 12:643069. doi: 10.3389/fpsyg.2021.643069 33716913 PMC7943442

[ref54] Zheng R. (1999). Psychological diagnosis of university students. Shandong: Shandong Education Press.

